# Systemic Immunomodulatory Effects of *Codonopsis pilosula* Glucofructan on S180 Solid-Tumor-Bearing Mice

**DOI:** 10.3390/ijms242115598

**Published:** 2023-10-26

**Authors:** Yuting Fan, Yan Long, Youshun Gong, Xiaoji Gao, Guoqiang Zheng, Haiyu Ji

**Affiliations:** 1 Center for Mitochondria and Healthy Aging, College of Life Sciences, Yantai University, Yantai 264005, China; fyting7766@163.com (Y.F.); 18282780162@163.com (Y.L.); gongys8991@163.com (Y.G.); xiaoji0405@163.com (X.G.); 2 Center for Functional Factors and Body Immune Regulation Research, College of Food Science and Engineering, Tianjin University of Science and Technology, Tianjin 300457, China; zheng@tust.edu.cn

**Keywords:** *Codonopsis pilosula* glucofructan, antitumor immune response, apoptosis mechanisms

## Abstract

The immune functions of the body are intricately intertwined with the onset and advancement of tumors, and immunotherapy mediated by bioactive compounds has exhibited initial effectiveness in overcoming chemotherapy resistance and inhibiting tumor growth. However, the comprehensive interpretation of the roles played by immunologic components in the process of combating tumors remains to be elucidated. In this study, the *Codonopsis pilosula* glucofructan (CPG) prepared in our previous research was employed as an immunopotentiator, and the impacts of CPG on both the humoral and cellular immunity of S180 tumor-bearing mice were investigated. Results showed that CPG administration of 100 mg/kg could effectively inhibit tumor growth in mice with an inhibitory ratio of 45.37% and significantly improve the expression of Interleukin-2 (IL-2), Interferon-γ (IFN-γ), and Tumor Necrosis Factor-α (TNF-α). Additionally, CPG clearly enhanced B-cell-mediated humoral immunity and immune-cell-mediated cellular immunity, and, finally, induced S180 cell apoptosis by arresting cells in the G0/G1 phase, which might result from the IL-17 signaling pathway. These data may help to improve comprehension surrounding the roles of humoral and cellular immunity in anti-tumor immune responses.

## 1. Introduction

Cancer has emerged as the most prevalent malignant disease, posing horrific threats to human health and life [1]. Chemotherapy is a contemporary therapeutic approach employed for the treatment of individuals with tumors [2]. However, its treatment efficacy is limited as cancer cells develop resistance to chemotherapy drugs [3]. Medications with few adverse effects cannot always specifically eradicate cancer cells, ultimately resulting in disease recurrence [4]. In recent years, immunotherapy mediated by diverse bioactive compounds has made remarkable progress in mitigating chemotherapy resistance while inhibiting tumor relapse and metastasis [5]. As reported, there are various natural compounds with immunomodulatory activities, such as polysaccharides and flavonoids. Fucoidans, sulfated polymers with L-fucose and sulfate ester groups, have demonstrated strong anti-inflammatory and immunomodulatory activities [6]. In a previous study, *C. pilosula* glucofructan (CPG) was prepared with (2 → 1)-β-D-Fru*f* and (1 →)-α-D-Glcp as the backbone branched by (2 → 6)-β-D-Fru*f* and exhibited immunoregulatory functions in macrophages in vitro [7]. It was employed as an immunopotentiator for this anti-tumor immunity investigation.

The host’s protection against cancer heavily relies on both humoral and cellular immunity [8]. Different immunotherapy regulation involves a range of active ingredients, including IL-2, IFN-γ, and TNF-α. IL-2 engages the immune response to tumors by differentiating and activating cytotoxic and helper T lymphocytes while also stimulating B cell division and antibody production [9]. IFN-γ has emerged as the primary cytokine associated with anti-tumor immune response via identifying and eradicating cancerous cells [10]. TNF-α, a versatile cytokine, plays a crucial role in treating local inflammation and cancer [11,12]. The involvement of different cytokines contributes to the strengthening of immune response and the eradication of cancer cells, either through direct or indirect mechanisms.

Humoral immunity is primarily mediated by B cells, and B cells can enhance anti-tumor immune responses through the production of tumor-specific antibodies and facilitation of antigen presentation [13]. IgG, the primary antibody in serum, plays a crucial role in anti-tumor and anti-infection immune responses [14,15]. Furthermore, the presence of the IgG antibody indicates a harmonized immune reaction [16]. The detection of acute infections can be effectively facilitated by the presence of antigen-specific IgM, whereas the role of non-specific IgM remains unclear except in its potential to respond to glycolipids [17]. The IgA antibody acts as a protective shield for human mucosal surfaces and plays a crucial role in maintaining the overall well-being of the body. Research has shown that inadequate levels of IgA may be associated with an increased risk of cancer [18]. Tumor-related antigens have the ability to induce proliferation of fully developed B cells, leading to the secretion of different types of immunoglobulins, which are subsequently discharged into the circulatory system [19,20,21]. However, the effects of these antibodies on cancer cells have not been systematically investigated.

Various studies demonstrate that numerous immune cells in the body are involved in antitumor immune response. T lymphocytes play crucial roles in identifying and eradicating tumor cells [22]. Macrophages fulfill vital functions in tumor formation, development, and cell apoptosis and can be categorized into these two main types: M1 macrophages (classically activated) and M2 macrophages (alternatively activated) [23]. Natural killer (NK) cells belong to the innate immune system and possess both anticancer and proinflammatory responses to tumor cells with rare off-target toxicities [24]. The literature has demonstrated that cellular immunity might be the key immune response that directly eliminates tumor cells.

Extensive research has covered various anti-tumor immunity, which increases the difficulty in drawing comparable and definitive conclusions. Furthermore, the relevant reports about the immunomodulatory mechanisms of CPG against tumors are still insufficient. The aim of this study was to investigate the impacts of CPG on both humoral and cellular immunity by employing the S180 tumor-bearing mouse model, with a particular emphasis on the systemic immunoregulatory interaction. This comprehensive investigation at both molecular and cellular levels is expected to provide a theoretical basis for immunotherapy approaches to cancers.

## 2. Results

### 2.1. Physiological Indicators of S180-Bearing Mice

**Figure 1:**
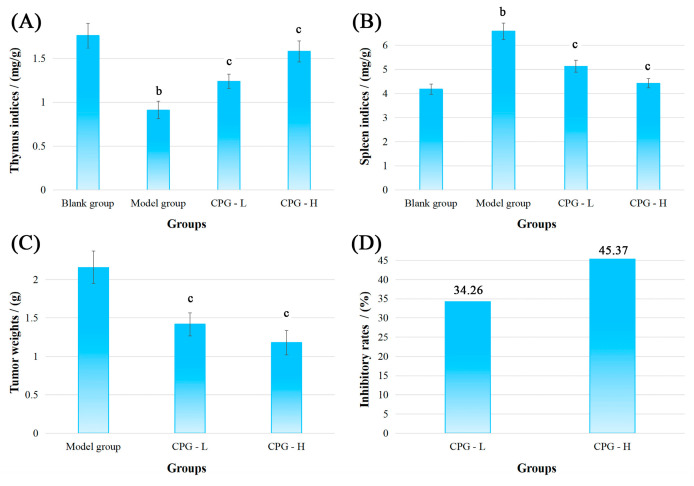
Physiological indicators of mice bearing S180 tumors after CPG treatments. (**A**): Thymus indices; (**B**): spleen indices; (**C**): tumor weights; (**D**): inhibitory rates. Note: ^b^ *p* < 0.05 compared with blank group; ^c^ *p* < 0.05 compared with model group.

The physiological indicators, namely thymus and spleen indices, tumor weights, and inhibitory rates, of S180-bearing mice in these groups were measured and calculated, and the results are shown in Figure 1. 

As presented in Figure 1A,B, compared with the blank group, the mice presented a significant reduction (*p* < 0.05) in thymus indices and an increase (*p* < 0.05) in spleen indices in the model group, indicating severe damage to the development of thymus and spleen under solid tumor attack [25]. However, noteworthy enhancements (*p* < 0.05) in thymus and spleen indices were discovered in CPG groups compared with the model group, suggesting that CPG could effectively protect immune organs against S180 tumors.

Figure 1C showed that the tumor weights in the model group were determined to be 2.16 ± 0.15 g after inoculation of S180 cells for 14 days. In comparison to the model group, the transplanted tumor weights were significantly decreased (*p* < 0.05) to 1.42 ± 0.15 g and 1.18 ± 0.10 g in CPG-L (50 mg/kg) and CPG-H (100 mg/kg) groups, respectively, with inhibitory rates of 34.26% and 45.37% (Figure 1D), demonstrating that CPG could remarkably suppress the proliferation of S180 solid tumors.

### 2.2. Cancer-Associated Cytokine Determination

**Figure 2:**
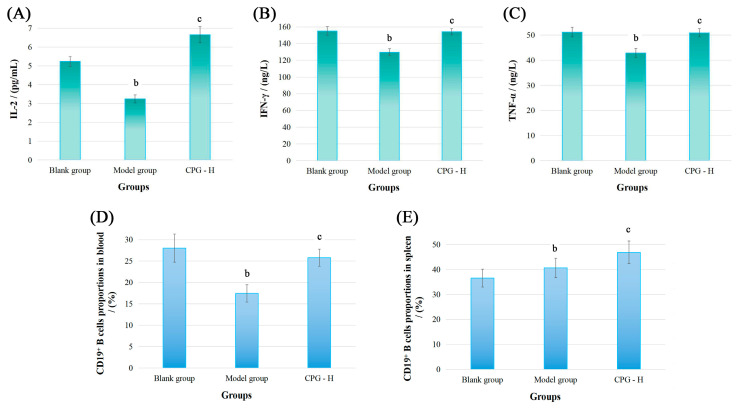
The cytokine levels in sera and CD19^+^ B cell proportions of tumor-bearing mice. (**A**): IL-2 levels; (**B**): IFN-γ levels; (**C**): TNF-α levels; (**D**): CD19^+^ B cell proportions in blood; (**E**): CD19^+^ B cell proportions in spleen. Note: ^b^ *p* < 0.05 compared with blank group; ^c^ *p* < 0.05 compared with model group.

Subsequently, the cancer-associated cytokine levels of IL-2, IFN-γ, and TNF-α in sera of these groups were determined, and relevant results are displayed in Figure 2A–C. 

As presented, the IL-2, IFN-γ, and TNF-α levels in mouse sera of the model group were all significantly reduced (*p* < 0.05) compared with the blank group. However, CPG administration demonstrated potent immunoregulatory effects by reinstating the cytokine balance in vivo, resulting in obviously improved expression (*p* < 0.05) of these cytokines compared with the model group, which indicated that CPG could remarkably enhance the systemic immunity of S180 tumor-bearing mice after considering physiological functions of these cytokines. Subsequently, the immunomodulatory abilities of CPG in S180 tumor-bearing mice were comprehensively elucidated from the perspectives of humoral immunity and cellular immunity.

### 2.3. Distributions of CD19^+^ B Cells

The CD19 molecule is a member of the immunoglobulin superfamily and serves as a specific surface marker of B cells in mammals. It functions primarily as a co-receptor influencing antigen selection, cell differentiation, and antibody expression levels [26,27]. The CD19^+^ B cell proportions were detected, and the results are presented in Figure 2D,E. As demonstrated, the CD19^+^ B cell percentages in peripheral blood of the model group were significantly decreased (*p* < 0.05) compared with the blank group. However, CPG treatment effectively improved the CD19^+^ B cell levels (*p* < 0.05) in tumor-bearing mice, indicating that tumor proliferation and CPG administration could significantly impact B cell proportions in peripheral blood. Additionally, the CD19^+^ B cell proportions in the model group were notably increased in mouse spleens (*p* < 0.05) compared with the blank group, while these proportions were obviously reduced relative to the CPG-H group (*p* < 0.05), suggesting that spleens might be responsible for humoral immune response, and the splenic B cell proportions are probably related to relevant antibody expression. 

### 2.4. Tumor-Specific IgG, IgM, and IgA Expression

**Figure 3:**
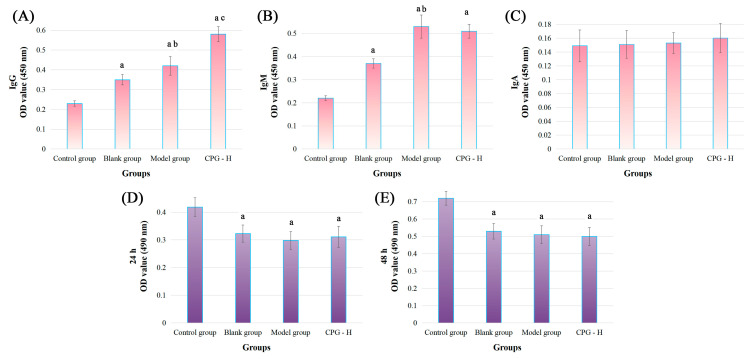
Tumor-specific antibody levels in mouse sera and the cytotoxic effects in vitro. (**A**): IgG levels; (**B**): IgM levels; (**C**): IgA levels; (**D**): cocultured for 24 h; (**E**): cocultured for 48 h. Note: ^a^ *p* < 0.05 compared with control group. ^b^ *p* < 0.05 compared with blank group. ^c^ *p* < 0.05 compared with model group.

Tumor-specific antibody expression of IgG, IgM, and IgA in sera was determined in order to further investigate the impacts of CPG on humoral immunity, and the results are shown in Figure 3A–C.

As presented, the tumor-specific antibody levels of IgG and IgM were significantly increased (*p* < 0.05) compared with the control group, indicating the reliable experimental procedures. The tumor-specific IgG and IgM antibody levels in the model group were remarkably increased (*p* < 0.05) compared with the blank group. Additionally, the CPG-H group exhibited considerably higher levels (*p* < 0.05) of the tumor-specific IgG antibody compared with the model group, which was different from the non-significant variation in the IgM antibody. Considering the tumor development processes in these groups, it could be deduced that IgM antibodies presented non-specific inhibitory effects on tumor cells, while IgG antibodies might play more critical roles in the clearance of tumor cells [28,29]. However, no correlation was observed between the IgA antibody in sera and tumor antigens in this study. 

### 2.5. Toxic Effects of Mouse Sera on S180 Cells

An MTT assay was applied to explore the direct cytotoxic effects of mouse sera on S180 cells in vitro, and the results are displayed in Figure 3D,E. As presented, compared with the control group, all sera treatments could apparently attenuate the S180 cell viabilities (*p* < 0.05), indicating that the antibodies in sera could directly inhibit the proliferation of tumor cells, which might be mainly induced by the IgM antibody according to variation trends of the above results. However, no notable disparities were determined between these groups, which confirmed again that the IgG antibody played specific and auxiliary roles in the process of anti-tumor immune responses, while the IgM antibody was mainly responsible for non-specific functions.

### 2.6. Coculture of S180 Cells and Sera

**Figure 4:**
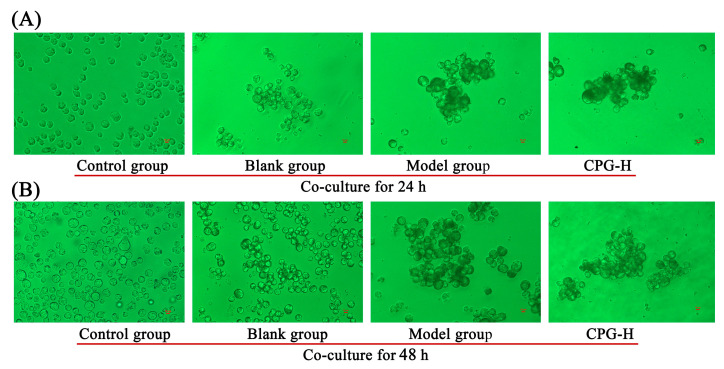
Morphological characterization of S180 tumor cells. (**A**): Cocultured for 24 h; (**B**): cocultured for 48 h.

The morphological changes in S180 cells after mouse sera treatments were determined after coculturing for 24 h and 48 h, as shown in Figure 4. As presented, apoptosis was not induced in these S180 cells after addition of mouse sera, while the cells were observed to be aggregated, and the presence of dark spots on cell surfaces was obvious in comparison with the control group. These findings suggested that partial antibodies could bind to the antigens of S180 cells, thereby decelerating the cell proliferation rates. The cell aggregation levels in both the model group and CPG-H group were significantly improved compared with the blank group, suggesting that the IgM antibody is responsible for the cells adhering to each other [30].

### 2.7. The Results of Blood Routine Examination

The blood routine examination of S180 tumor-bearing mice was conducted to explore the impacts of CPG on the peripheral blood parameters, and the results are presented in Table 1. 

Results showed that the growth of S180 cells in vivo significantly increased the numbers of platelets and leucocytes, especially the neutrophile granulocytes, while reducing the quantities of lymphocytes, erythrocytes and hemoglobin (*p* < 0.05), which indicated that the mice in the model group exhibited certain indications of inflammation and anemia. CPG administration could effectively increase the proportion of lymphocytes (*p* < 0.05) and relieve the negative impacts on peripheral blood induced by solid tumors. These results showed that the S180 cell proliferation in vivo demonstrated significant impacts on several indicators in peripheral blood, and the interventions targeting immune functions may be beneficial for cancer-induced anemia or other related symptoms [31,32].

### 2.8. Immune Cell Activities

The activities of macrophages, lymphocytes, and NK cells were assessed to further elucidate the impacts of CPG on cellular immunity, and the relevant results are presented in Table 2.

As presented, the activities of these three immune cells, namely macrophages, lymphocytes, and NK cells, exhibited a similar trend among these groups. The malignant proliferation of S180 cells in the model group showed significant inhibitory effects on these immune cell functions. Specifically, compared with the blank group, the pinocytosis abilities of macrophages were remarkably reduced in the model group, suggesting impaired macrophage response to tumor growth. Furthermore, the proliferation capacities of lymphocytes (T cells and B cells) were also significantly decreased in the model group, indicating the attenuated adaptive immunity through recognizing and eliminating cancerous or infected cells in tumor-bearing mice. Additionally, the NK cell activities were notably diminished in the model group compared with the blank group, suggesting the weakened ability to directly kill cancer cells. However, after CPG administration, there were significant improvements observed in all these immune cell activities compared with the model group, indicating that CPG demonstrated strong immune enhancement properties in these immune cells, specifically targeting S180 tumor cells [33].

### 2.9. Cell Cycle Distributions and Apoptotic Rates in Tumors

**Figure 5:**
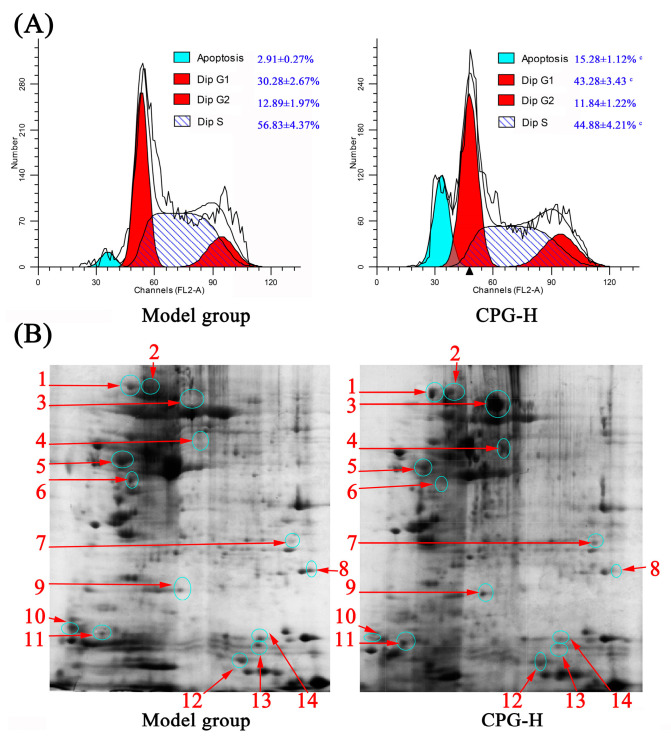
Cell cycle distribution (**A**) and 2-DE results (**B**) of solid tumor cells in mice. Note: ^c^ *p* < 0.05 compared to model group. 1~14 represent serial numbers of 14 proteins with differential expression.

The distributions of cell cycle and apoptotic rates of solid tumor cells in mice were determined, and the results are presented in Figure 5A. As shown, the cell percentages in the G0/G1 phase and apoptotic rates in solid tumors of CPG-treated mice were obviously increased (*p* < 0.05) compared with the model group, whereas the proportions in the S phase were remarkably reduced (*p* < 0.05), which indicated that the growth of S180 tumor cells in vivo was effectively inhibited by CPG-enhanced cellular immune response. These findings provided valuable insights into potential therapeutic strategies for cancer treatment [34]. 

### 2.10. Differences in Protein Expression of Solid Tumors in Each Group

Two-dimensional gel electrophoresis (2-DE) and liquid chromatography-mass spectrometry (LC-MS) were performed to further analyze the effects of CPG on protein expression of solid tumor cells, as shown in Figure 5B. As shown, there were a total of 14 differential proteins determined in the experiment. The identification of these proteins would provide valuable insights into understanding the underlying mechanisms and potential biomarkers associated with anti-tumor immunity.

The separated proteins were stained with colloidal Coomassie brilliant blue G-250, and the differently expressed proteins were determined via gray values of protein spots and subsequently identified via LC-MS.

The information of 14 differential proteins is shown in Table 3.

As shown, there were seven kinds of proteins highly expressed in the model group, namely vimentin, annexin A5, phosphoglycerate mutase 2, prostaglandin E synthase 3, NPC intracellular cholesterol transporter 2, protein S100-A9, and small ubiquitin-related modifier 1. These highly expressed proteins revealed interesting insights into their potential roles in cancer progression. Vimentin is a cytoskeletal protein implicated in tumor invasion and metastasis; annexin A5 is involved in cell membrane repair and apoptosis regulation; phosphoglycerate mutase 2 plays a role in glycolysis and energy metabolism; prostaglandin E synthase 3 is an enzyme involved in inflammation and pain-signaling pathways; NPC intracellular cholesterol transporter 2 is important for cholesterol homeostasis and lipid metabolism; protein S100-A9 is linked to inflammatory responses and immune system activation; and small ubiquitin-related modifier 1 (SUMO-1) regulates protein function through post-translational modification. These highly expressed proteins might contribute to cancer incidence and development and are potential targets for therapeutic intervention or biomarkers for diagnosis/prognosis assessment. 

Moreover, seven differential upregulated proteins in the CPG-H group were identified as heat shock protein (HSP) 90-alpha, heat shock protein (HSP) 90-beta, albumin, 60 kDa heat shock protein, carbonic anhydrase 3, osteoclast-stimulating factor 1 and myosin light polypeptide. The upregulation of these proteins suggested that CPG treatment could exhibit positive effects on various physiological processes such as stress responses (HSPs), bone metabolism (osteoclast-stimulating factor), muscle function (myosin light polypeptide), and acid-base balance regulation (carbonic anhydrase). Overall, these findings provided valuable insights into the potential therapeutic benefits of CPG treatment on tumor-bearing mice.

**Figure 6:**
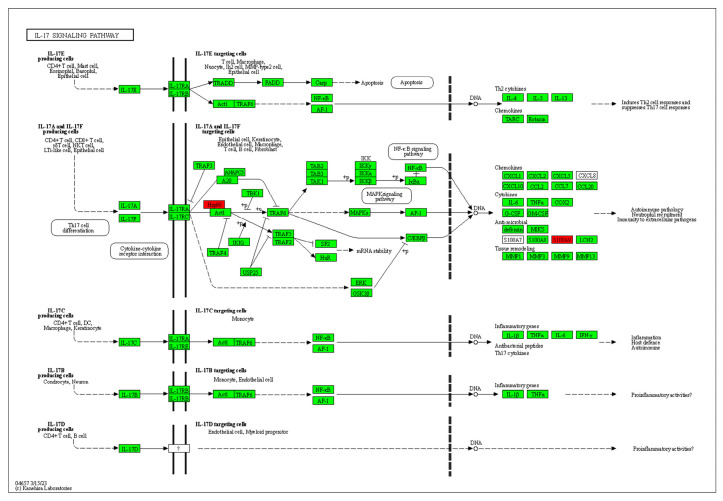
Diagram of IL-17 signaling pathway. Note: Proteins with red background are detected proteins in Table 3.

The pathway enrichment tool KOBAS 3.0 was used to analyze these 14 differential proteins online, and *p* < 0.001 and corrected *p* < 0.01 were used as the criteria for screening. Finally, the IL-17 signaling pathway was selected as the most likely signaling pathway and is shown in Figure 6. Cytokines of the IL-17 family have been reported to possess a diverse array of immunomodulatory functions, potentially attributed to their capacity to induce numerous immune signaling molecules. IL-17 is primarily responsible for triggering and controlling inflammatory reactions and allergic responses [35] via stimulating the production of various cytokines (such as IL-6 and TNF-α) and chemokines (including IL-8 and MCP-1) from multiple cells. IL-17 also plays a crucial role in the activity regulation of T-helper 17 (Th 17) cells. Consequently, the involvement of the IL-17 family has been implicated in numerous immune/autoimmune-related diseases including anti-tumor immunity [36,37].

## 3. Discussion

### 3.1. Systemic Immunity and Anti-Tumor Effects

The body immune function is intricately intertwined with the occurrence and progression of tumors [38]. The decline or inhibition in the body’s immunity would weaken the suppression and elimination of tumor cells, thereby leading to an increased incidence of cancer cells, while the recognition of tumor antigens would trigger the immunological reaction against tumor cells [39]. Immune responses can be divided into humoral and cellular immunity, which can also be classified as specific or non-specific immunity [40]. However, the contents of existing studies on the immune system’s roles in anti-tumor responses are scattered and inconsistent, which increases the difficulty of achieving a comprehensive understanding of the relationship between the immune system and tumors.

### 3.2. Systemic Immunity Potential of CPG

*C. pilosula* is a well-known herbaceous perennial in Northern China, known for its health benefits and ginseng-like properties, and the polysaccharide components have demonstrated diverse biological functions, including anti-tumor and immunoregulatory activities [41,42]. However, the adopted extraction methods could significantly impact the yield, properties, and bioactivities of polysaccharides [43]. In our previous study, water-soluble *C. pilosula* glucofructan (CPG) was prepared and presented immunoregulatory function in vitro [7], which was consistent with relevant research [44]. The primary objective of this study was investigating the comprehensive immunoregulatory activity, encompassing both humoral and cellular immunity, of CPG in S180 tumor-bearing mice.

### 3.3. Antitumor Effects of CPG

Physiological indicators are the most fundamental and intuitive for assessing the intervention effects of experimental substances on tumor-bearing mice [45]. IL-2, IFN-γ, and TNF-α are key cytokines that play pivotal roles in the immune response to cancer by activating various immune cells and promoting tumor cell apoptosis [46,47]. In this study, the CPG administration of 100 mg/kg significantly reduced tumor weights in mice with an inhibitory ratio of 45.37% and obviously improved the expression levels of cytokines such as IL-2, IFN-γ, and TNF-α in tumor-bearing mice, thereby bolstering the systemic immune responses to tumors.

### 3.4. CPG-Enhanced Anti-Tumor Humoral Immunity

The expression of IL-2R in B cells could be enhanced by IL-2, thereby inducing immunoglobulin production and promoting antigen phagocytosis [48]. IFN-γ has the potential to augment immunoglobulin secretion in activated B cells and facilitate the B cells progression into the S phase of the cell cycle. TNF-α may serve as a co-stimulatory factor for mitogen-activated normal B cells [49]. The results of the MTT assay indicated that the mouse sera from each group did not exhibit specific inhibitory effects on tumor cells in vitro. Combined with the analysis of splenic B cell distributions and sera antibody levels, it can be speculated that IgM and IgG antibodies presented non-specific and specific inhibitory effects on tumor cells, which might play auxiliary roles in anti-tumor cellular immune responses in vivo.

### 3.5. CPG-Enhanced Anti-Tumor Cellular Immunity

As reported, macrophages are the predominant immune cells in the tumor microenvironment, and the polarization towards the tumoricidal M1 phenotype would be advantageous for efficient elimination of tumor cells in vivo [50]. The T lymphocytes can eliminate malignant cells through synaptic exocytosis of cytotoxic granules or secretion of cytokines [51]. The NK cells exhibit important roles in the suppression of cancer cells mediated by the perforin-granzyme B signaling pathway [52]. In this study, CPG administration remarkably enhanced these cells capacities and attenuated the side effects such as anemia and inflammation caused by tumor proliferation, suggesting a strengthened anticancer cellular immunity in S180 tumor-bearing mice.

### 3.6. Solid Tumor Cell Apoptosis Induced by CPG-Enhanced Immunity

Chemotherapy is the primary therapeutic approach for a wide range of solid tumors, and chemical agents can effectively induce caspase-dependent apoptosis in cancer cells, finally resulting in the effective control of solid tumor growth [53]. The underlying apoptosis mechanisms in solid tumor cells induced by anti-tumor immune responses are highly intricate and extensive. However, two-dimensional electrophoresis and proteomic analysis continue to serve as crucial screening tools and primary qualitative methods [54]. In the present study, compared with the model group, there were a total of 14 differential proteins identified in solid tumors of CPG-treated mice, with 7 proteins downregulated and 7 other proteins highly expressed, and relevant analysis indicated that the IL-17 signaling pathway is the most likely to be involved in CPG-mediated antitumor cellular immunity.

## 4. Materials and Methods

### 4.1. Materials

CPG was isolated from *C. pilosula* using the water-extraction (liquid–material ratio of 33 mL/g) and alcohol-precipitation (Ethanol volume fraction of 75%) methods and purified via dialysis (molecular weight cut-off of 1000 Da) with tap water and sephadex-G25 column chromatography. Mouse sarcoma S180 cells were provided by the Shanghai Institute of Biochemistry and Cell Biology (Shanghai, China). Dimethyl sulfoxide (DMSO), 3-(4,5-dimethylthiazol-2-yl)-2,5diphenyltetrazolium bromide (MTT), and cell cycle detection kits were purchased from Beijing Solarbio Science & Technology Co., Ltd (Beijing, China). IL-2, IFN-γ, and TNF-α cytokine ELISA kits were obtained from the Nanjing Jiancheng Bioengineering Institute (Nanjing, China). CD19-PE antibodies were provided by BioLegend (San Diego, CA, USA). Anti-mouse IgG/HRP, IgM/HRP, and IgA/HRP antibodies were acquired from the Bioss Biotechnology Company (Beijing, China). Other agents employed were of analytical quality.

### 4.2. Animals and Cell Lines

Forty female Kunming mice (6 weeks, 20.0 ± 2.0 g) were purchased from SPF (Beijing) Biotechnology Co., LTD (Beijing, China). The animals were reared in a meticulously controlled environment, free from any pathogens, maintaining a consistent temperature of 23 ± 2 °C and a relative humidity of 50 ± 5%. They strictly adhered to a well-regulated light/dark cycle spanning over 12 h while enjoying uninterrupted access to a standardized pellet diet and tap water throughout the entire duration of the experiment. All animal-related procedures were approved by the Local Ethics Committee for Animal Care and carried out at Tianjin University of Science and Technology.

The animals were randomly divided into four groups, namely the blank group, model group, and CPG groups (50 mg/kg and 100 mg/kg). During the experiment, both the blank and model groups were administered saline solution. CPG groups were given polysaccharides with 50 mg/kg (CPG-L) and 100 mg/kg (CPG-H) per day. After 7 days of oral administration, all the mice were transplanted with S180 cells in the right forelimb armpit using a volume of 0.2 mL containing 5 × 10^6^ cells, except for the blank group. Finally, all groups were sacrificed to provide samples for further research after administration for an additional 14 days.

### 4.3. Immune Organ Indices and Tumor Weights

The immune organ indices were evaluated according to the following formula: Organ indices (%) = average weights of organs/(average body weights) × 100 [55]. The formula provided below was utilized to determine the inhibitory rates: Inhibitory rates (%) = (M_α_ − M_β_)/M_α_ × 100, where M_α_ and M_β_ represent the mean tumor weights of the model group and CPG groups, respectively [56].

### 4.4. IL-2, IFN-γ, and TNF-α Determination

The expression of IL-2, IFN-γ, and TNF-α levels in mouse sera was assessed by employing the corresponding ELISA kits, and the results were obtained using a BioRad Model 680 Microplate Reader (Hercules, CA, USA) at 450 nm [57].

### 4.5. CD19^+^ B Cell Distributions

The CD19^+^ B cell distributions and proportions in blood and spleens were determined using flow cytometry equipment (Bergen, NJ, USA). After depletion of red blood cells, 2 μL of PE-CD19 antibody was added to each blood and spleen cell tube for 20 min incubation. Subsequently, unbound antibodies were eliminated by PBS washing 3 times. Finally, the samples were determined via flow cytometry analysis using FL2 channel detection, and the results were analyzed using BD CellQuest^TM^ Pro software (version 6.0).

### 4.6. S180-Specific IgG, IgM, and IgA Level Determination

The S180 cells were resuspended using ELISA coating buffer and inoculated in 96-well plates, then incubated overnight at 4 °C to immobilize the tumor antigen on the microplate surface. After removing the coating solution, the plates were treated with blocking buffer at 37 °C for 1 h to enhance antigen specificity. Mouse sera of 100 μL (1:200 dilution with PBS) from each group were added and incubated at 37 °C for 1 h to allow antibodies to bind to antigens. Subsequently, HRP-labeled anti-mouse IgG, IgM, and IgA antibodies of 100 μL (1:200 dilution with PBS) were added and incubated at 37 °C for another 1 h for to bind secondary antibodies to primary antibodies. Finally, color development solution and termination solution were added before measuring absorbance at 450 nm using a BioRad 680 microplate reader (CA, USA).

### 4.7. Tumor Cell Viability Detection

The MTT assay was employed to assess the differential cytotoxicity of mouse sera on S180 cells among various groups. Initially, S180 cells were adjusted to an appropriate density (2 × 10^5^ cells/mL) and seeded in 96-well plates with 100 μL/well. Then, all groups were treated with physiological concentrations of sera (10%) except the control group. Following coculture periods of 24 h and 48 h, MTT reagent (dissolved in culture medium with a concentration of 5 mg/mL) of 10 μL was added and incubated for 4 h. Subsequently, the culture medium of 150 μL was removed, followed by addition of 150 μL DMSO solution. The absorbance at 490 nm was measured using a microplate reader after thorough shaking [58].

### 4.8. Morphology of Sera-Treated S180 Cells 

The cell culture procedure remained consistent with the description in Section 2.7. Prior to the addition of MTT reagent, these cells were subjected to microscopic examination and imaging via an inverted fluorescence microscope (Nikon, Tokyo, Japan).

### 4.9. Blood Routine Parameter Examination

The mouse blood samples of 50 μL were collected and treated with EDTA-K2 reagent for anticoagulation to prevent thrombosis. Subsequently, the blood samples were sequentially analyzed using an XFA-6130 automatic blood analyzer (Nanjing, China), followed by data organization and analysis [59].

### 4.10. Immune Cell Activity Detection

Referring to the published methods, splenic T cells and B cells were stimulated with ConA and LPS as mitogens, and the proliferative activities of T cells and B cells were assessed using the MTT assay. Additionally, the cytotoxicity of splenic natural killer (NK) cells against S180 target cells was measured, while the pinocytosis ability of peritoneal macrophages was analyzed using neutral red solution as an indicator [60].

### 4.11. Cell Cycle Distribution Determination

Solid tumor tissues of appropriate size were excised, and tumor cell suspensions from different groups were prepared, then fixed in pre-chilled 75% ethanol, and stored under 4 °C overnight. After washing three times with PBS to eliminate ethanol, 50 μL propidium iodide (PI) and RNAse working solution were added for 30 min incubation in darkness. The final samples were subsequently detected and analyzed using flow cytometry equipment (Bergen, NJ, USA).

### 4.12. 2D Gel Electrophoresis

Two-dimensional electrophoresis (2-DE) coupled with liquid chromatography–tandem mass spectrometry (LC-MS/MS) was employed to effectively and intuitively analyze the variations in S180 solid tumors protein expression between the model group and CPG-H group. Initially, protein samples from the solid tumors were prepared using a solution for two-dimensional electrophoresis sample preparation and immobilized on dry tape for isoelectric focusing. The voltage for isoelectric focusing (IEF) was set as follows: 50 V for 2.5 h, 100 V for 0.5 h, 500 V for 1 h, and then increased to 1000 V for another 1 h before being held at 8000 V for an additional 3 h. Subsequently, focused proteins were analyzed by running 12% SDS-PAGE gel at a current of 30 mA/gel for 1 h followed by an increase to 90 mA/gel for another 2 h. Finally, colloidal Coomassie brilliant blue staining was utilized to facilitate visual analysis, and differential proteins were extracted for further qualitative identification through LC-MS.

### 4.13. Statistical Analysis

The experimental data for each treatment are presented as the mean ± S.D., and statistical significance was assessed using either one-way ANOVA or Student’s *t*-test. A *p*-value below 0.05 was deemed to indicate statistical significance.

## 5. Conclusions

In conclusion, CPG administration could remarkably inhibit the transplanted tumor growth in mice with an inhibitory ratio of 45.37% and significantly improve the expression of IL-2, IFN-γ, and TNF-α. Subsequent results demonstrated that CPG could obviously enhance the B-cell-mediated humoral immunity and immune-cell-mediated cellular immunity, finally inducing S180 cell apoptosis via arresting cells in the G0/G1 phase, which might have resulted from the IL-17 signaling pathway. However, further studies are still needed to investigate the activation mechanisms of cellular immune responses and apoptotic mechanisms in solid tumors.

## Figures and Tables

**Table 1:** Results of blood routine examination of mice in each group.

Measurement	Unit	Reference Value	Blank Group	Model Group	CPG-H Group
Leucocyte count	10^9^/L	4–12	6.97 ± 1.26	13.53 ± 1.46 ^b^	9.33 ± 1.53 ^c^
Lymphocyte percentage	%	54–85	76.31 ± 5.24	34.08 ± 2.21 ^b^	65.37 ± 4.42 ^c^
Intermediate cell percentage	%	0–9	1.35 ± 0.22	1.59 ± 0.23	1.62 ± 0.24
Neutrophil percentage	%	12–44	22.32 ± 2.13	64.34 ± 3.21 ^b^	33.03 ± 2.65 ^c^
Erythrocyte count	10^12^/L	6–12.5	8.81 ± 0.53	7.42 ± 0.52 ^b^	8.70 ± 0.63 ^c^
Hemoglobin	g/L	100–190	173.35 ± 9.83	137.68 ± 8.99 ^b^	166.43 ± 9.21 ^c^
Mean corpuscular volume	fL	51–65	58.70 ± 4.83	59.00 ± 4.35	58.46 ± 4.13
Hematocrit	L/L	0.35–0.55	0.47 ± 0.03	0.40 ± 0.02	0.47 ± 0.03
Mean corpuscular hemoglobin	Pg	12–30	21.80 ± 1.83	20.13 ± 0.99	21.23 ± 1.12
Mean corpuscular hemoglobin concentration	g/L	230–330	373.30 ± 21.12	342.95 ± 24.56 ^b^	364.75 ± 23.47 ^c^
Red cell distribution width	fL	15–55	42.41 ± 4.56	41.94 ± 3.96	42.41 ± 5.12
Platelet count	10^9^/L	100–300	333.40 ± 14.26	455.21 ± 21.34 ^b^	368.32 ± 22.88 ^c^
Mean platelet volume	fL	6.1–12	7.42 ± 1.56	7.93 ± 1.52	7.98 ± 1.82
Plateletcrit	L/L	0.1–0.6	0.23 ± 0.01	0.33 ± 0.03 ^b^	0.27 ± 0.02 ^c^
Platelet distribution width	%	1–30	14.89 ± 1.21	14.89 ± 1.42	14.87 ± 1.31

Note: ^b^ *p* < 0.05 compared with blank group; ^c^ *p* < 0.05 compared with model group.

**Table 2:** Effects of CPG on immune cell activities.

Group	Macrophage Pinocytosis	Lymphocyte Proliferation Capacity		NK Cell Killing Activity
	OD_550 nm_ Value	Canavalin A (Con A)	Lipopolysaccharide (LPS)	(%)
Blank group	0.76 ± 0.04	4.20 ± 0.35	6.09 ± 0.31	60.52 ± 4.05
Model group	0.31 ± 0.02 ^b^	2.47 ± 0.13 ^b^	3.95 ± 0.23 ^b^	36.13 ± 2.08 ^b^
CPG-H group	0.71 ± 0.05 ^c^	3.87 ± 0.29 ^c^	5.49 ± 0.37 ^c^	63.31 ± 3.79 ^c^

Note: ^b^ *p* < 0.05 compared with blank group. ^c^ *p* < 0.05 compared with model group.

**Table 3:** A list of differently expressed proteins identified through LC-MS.

No.	Accession	Name	Nominal Mass (Mr)	Calculated pI Value	Expression Compared with Model Group
1	P07901	Heat shock protein HSP 90-alpha	85,134	4.97	Upregulation
2	P11499	Heat shock protein HSP 90-beta	83,571	4.97	Upregulation
3	P07724	Albumin	70,700	5.75	Upregulation
4	P63038	60 kDa heat shock protein	61,088	5.91	Upregulation
5	P20152	Vimentin	53,712	5.06	Downregulation
6	P48036	Annexin A5	35,787	4.83	Downregulation
7	P16015	Carbonic anhydrase 3	29,633	6.89	Upregulation
8	O70250	Phosphoglycerate mutase 2	28,980	8.65	Downregulation
9	Q62422	Osteoclast-stimulating factor 1	23,996	5.46	Upregulation
10	Q9R0Q7	Prostaglandin E synthase 3	18,995	4.36	Downregulation
11	Q60605	Myosin light polypeptide	16,930	4.40	Upregulation
12	Q9Z0J0	NPC intracellular cholesterol transporter 2	16,774	7.59	Downregulation
13	P31725	Protein S100-A9	13,211	6.64	Downregulation
14	P63166	Small ubiquitin-related modifier 1	11,607	5.35	Downregulation

## Data Availability

Data sharing not applicable.

## References

[1] Breous E., Thimme R. (2011). Potential of immunotherapy for hepatocellular carcinoma. J. Hepatol..

[2] Sarkar F.H., Li Y. (2006). Using chemopreventive agents to enhance the efficacy of cancer therapy. Cancer Res..

[3] Wang J., Liu W., Chen Z., Chen H. (2017). Physicochemical characterization of the oolong tea polysaccharides with high molecular weight and their synergistic effects in combination with polyphenols on hepatocellular carcinoma. Biomed. Pharmacother..

[4] Kareva I., Waxman D.J., Lakka Klement G. (2015). Metronomic chemotherapy: An attractive alternative to maximum tolerated dose therapy that can activate anti-tumor immunity and minimize therapeutic resistance. Cancer Lett..

[5] Yu J., Dong X.-d., Jiao J.-s., Ji H.-y., Liu A.-j. (2021). Antitumor and immunoregulatory activities of a novel polysaccharide from Astragalus membranaceus on S180 tumor-bearing mice. Int. J. Biol. Macromol..

[6] Eswar K., Mukherjee S., Ganesan P., Rengan A.K. (2023). Immunomodulatory natural polysaccharides: An overview of the mechanisms involved. Eur. Polym. J..

[7] Ji H.-Y., Yu J., Jiao J.-S., Dong X.-D., Yu S.-S., Liu A.-J. (2022). Ultrasonic-Assisted Extraction of Codonopsis pilosula Glucofructan: Optimization, Structure, and Immunoregulatory Activity. Nutrients.

[8] Suckow M.A. (2013). Cancer vaccines: Harnessing the potential of anti-tumor immunity. Vet. J..

[9] Rossowska J., Pajtasz-Piasecka E., Rysnik O., Wojas J., Krawczenko A., Szyda A., Dus D. (2011). Generation of antitumor response by IL-2-transduced JAWS II dendritic cells. Immunobiology.

[10] Kursunel M.A., Esendagli G. (2016). The untold story of IFN-gamma in cancer biology. Cytokine Growth Factor. Rev..

[11] Mausner-Fainberg K., Regev K., Kolb H., Vaknin-Dembinsky A., Karni A. (2015). Increased neutralization capacity of TNF-alpha in sera of relapsing remitting multiple sclerosis patients is not related to soluble TNF-alpha receptors or anti-TNF-alpha autoantibody levels. J. Neuroimmunol..

[12] Basappa, Murugan S., Kavitha C.V., Purushothaman A., Nevin K.G., Sugahara K., Rangappa K.S. (2010). A small oxazine compound as an anti-tumor agent: A novel pyranoside mimetic that binds to VEGF, HB-EGF, and TNF-alpha. Cancer Lett..

[13] Qin X., Yang T., Xu H., Zhang R., Zhao S., Kong L., Yang C., Zhang Z. (2023). Dying tumor cells-inspired vaccine for boosting humoral and cellular immunity against cancer. J. Control. Release.

[14] Khanna D., Rana P.S. (2017). Multilevel ensemble model for prediction of IgA and IgG antibodies. Immunol. Lett..

[15] Breen L.D., Pucic-Bakovic M., Vuckovic F., Reiding K., Trbojevic-Akmacic I., Srajer Gajdosik M., Cook M.I., Lopez M.J., Wuhrer M., Camara L.M. (2016). IgG and IgM glycosylation patterns in patients undergoing image-guided tumor ablation. Biochim. Biophys. Acta.

[16] Dash S.K., Chattopadhyay S., Tripathy S., Dash S.S., Das B., Mandal D., Mahapatra S.K., Bag B.G., Roy S. (2015). Self-assembled betulinic acid augments immunomodulatory activity associates with IgG response. Biomed. Pharmacother..

[17] Chapey E., Wallon M., Peyron F. (2017). Evaluation of the LDBIO point of care test for the combined detection of toxoplasmic IgG and IgM. Clin. Chim. Acta.

[18] Ludvigsson J.F., Neovius M., Ye W., Hammarström L. (2015). IgA Deficiency and Risk of Cancer: A Population-Based Matched Cohort Study. J. Clin. Immunol..

[19] Vos Q., Lees A., Wu Z.Q., Snapper C.M., Mond J.J. (2000). B-cell activation by T-cell-independent type 2 antigens as an integral part of the humoral immune response to pathogenic microorganisms. Immunol. Rev..

[20] Selter R.C., Biberacher V., Grummel V., Buck D., Eienbroker C., Oertel W.H., Berthele A., Tackenberg B., Hemmer B. (2013). Natalizumab treatment decreases serum IgM and IgG levels in multiple sclerosis patients. Mult. Scler..

[21] Diaz-Zaragoza M., Hernandez-Avila R., Govezensky T., Mendoza L., Meneses-Ruiz D.M., Ostoa-Saloma P. (2015). Comparison patterns of 4 T1 antigens recognized by humoral immune response mediated by IgG and IgM antibodies in female and male mice with breast cancer using 2D-immnunoblots. Immunobiology.

[22] Figueroa C., Galvez-Cancino F., Oyarce C., Contreras F., Prado C., Valeria C., Cruz S., Lladser A., Pacheco R. (2017). Inhibition of dopamine receptor D3 signaling in dendritic cells increases antigen cross-presentation to CD8+ T-cells favoring anti-tumor immunity. J. Neuroimmunol..

[23] Ochioni A.C., Imbroisi Filho R., Esteves A.M., Leandro J.G.B., Demaria T.M., do Nascimento Júnior J.X., Pereira-Dutra F.S., Bozza P.T., Sola-Penna M., Zancan P. (2021). Clotrimazole presents anticancer properties against a mouse melanoma model acting as a PI3K inhibitor and inducing repolarization of tumor-associated macrophages. Biochim. Et Biophys. Acta (BBA)-Mol. Basis Dis..

[24] Naeimi Kararoudi M., Tullius B.P., Chakravarti N., Pomeroy E.J., Moriarity B.S., Beland K., Colamartino A.B.L., Haddad E., Chu Y., Cairo M.S. (2020). Genetic and epigenetic modification of human primary NK cells for enhanced antitumor activity. Semin. Hematol..

[25] Ji H.-y., Dai K.-y., Liu C., Yu J., Liu A.-j., Chen Y.-f. (2022). The ethanol-extracted polysaccharide from Cynanchum paniculatum: Optimization, structure, antioxidant and antitumor effects. Ind. Crops Prod..

[26] Morbach H., Schickel J.N., Cunningham-Rundles C., Conley M.E., Reisli I., Franco J.L., Meffre E. (2016). CD19 controls Toll-like receptor 9 responses in human B cells. J. Allergy Clin. Immunol..

[27] van Zelm M.C., Bartol S.J., Driessen G.J., Mascart F., Reisli I., Franco J.L., Wolska-Kusnierz B., Kanegane H., Boon L., van Dongen J.J. (2014). Human CD19 and CD40L deficiencies impair antibody selection and differentially affect somatic hypermutation. J. Allergy Clin. Immunol..

[28] Ostoa-Saloma P., Morales-Montor J., Segovia-Mendoza M. (2021). Chapter 6—The IgM as a tool for recognition of early tumoral antigens. Immunotherapy in Resistant Cancer: From the Lab Bench Work to Its Clinical Perspectives.

[29] Yang S., Cui M., Liu Q., Liao Q. (2022). Glycosylation of immunoglobin G in tumors: Function, regulation and clinical implications. Cancer Lett..

[30] Jones P.C. (2013). Does a “thiol shield” protect tumors from natural IgM antibody, and, if so, how can it be suppressed?. Med. Hypotheses.

[31] Humar R., Schaer D.J., Vallelian F. (2022). Erythrophagocytes in hemolytic anemia, wound healing, and cancer. Trends Mol. Med..

[32] Khusnurrokhman G., Wati F.F. (2022). Tumor-promoting inflammation in lung cancer: A literature review. Ann. Med. Surg..

[33] Dong X.-d., Liu Y.-n., Zhao Y., Liu A.-j., Ji H.-y., Yu J. (2021). Structural characterization of a water-soluble polysaccharide from Angelica dahurica and its antitumor activity in H22 tumor-bearing mice. Int. J. Biol. Macromol..

[34] Ji H., Lou X., Jiao J., Li Y., Dai K., Jia X. (2023). Preliminary Structural Characterization of Selenium Nanoparticle Composites Modified by Astragalus Polysaccharide and the Cytotoxicity Mechanism on Liver Cancer Cells. Molecules.

[35] Liu L., Liu R., Wei C., Li D., Gao X. (2023). The role of IL-17 in lung cancer growth. Cytokine.

[36] Cebi M., Cakar A., Erdogdu E., Durmus-Tekce H., Yegen G., Ozkan B., Parman Y., Saruhan-Direskeneli G. (2023). Thymoma patients with or without myasthenia gravis have increased Th17 cells, IL-17 production and ICOS expression. J. Neuroimmunol..

[37] Seif F., Torki Z., Zalpoor H., Habibi M., Pornour M. (2023). Breast cancer tumor microenvironment affects Treg/IL-17-producing Treg/Th17 cell axis: Molecular and therapeutic perspectives. Mol. Ther.—Oncolytics.

[38] McIntyre C.L., Temesgen A., Lynch L. (2023). Diet, nutrient supply, and tumor immune responses. Trends Cancer.

[39] Yang K., Chen J., Chen J., Wang Z., Song B., Li R., Zhong S., Cheong K.-L. (2023). The effect mechanism of polysaccharides inhibit tumor immune escape: A review. J. Funct. Foods.

[40] Jiménez-Chávez Á.d.J., Nava-García B.K., Bustos-Jaimes I., Moreno-Fierros L. (2021). B19-VLPs as an effective delivery system for tumour antigens to induce humoral and cellular immune responses against triple negative breast cancer. Immunol. Lett..

[41] Yu J., Dong X.-d., Jiao J.-s., Yu S.-s., Ji H.-y., Liu A.-j., Chen Y. (2022). Extraction, purification, and biological activities in vivo of a novel fructose-rich polysaccharide from Codonopsis pilosula. Ind. Crops Prod..

[42] Li N., Xiong Y.X., Ye F., Jin B., Wu J.J., Han M.M., Liu T., Fan Y.K., Li C.Y., Liu J.S. (2023). Isolation, Purification, and Structural Characterization of Polysaccharides from Codonopsis pilosula and Their Anti-Tumor Bioactivity by Immunomodulation. Pharmaceuticals.

[43] Bai R.-B., Zhang Y.-J., Fan J.-M., Jia X.-S., Li D., Wang Y.-P., Zhou J., Yan Q., Hu F.-D. (2020). Immune-enhancement effects of oligosaccharides from Codonopsis pilosula on cyclophosphamide induced immunosuppression in mice. Food Funct..

[44] Sun Q.-L., Li Y.-X., Cui Y.-S., Jiang S.-L., Dong C.-X., Du J. (2019). Structural characterization of three polysaccharides from the roots of Codonopsis pilosula and their immunomodulatory effects on RAW264.7 macrophages. Int. J. Biol. Macromol..

[45] Zhang H., Dong X., Ji H., Yu J., Liu A. (2023). Preparation and structural characterization of acid-extracted polysaccharide from Grifola frondosa and antitumor activity on S180 tumor-bearing mice. Int. J. Biol. Macromol..

[46] Lee H.K., Nam M.-W., Go R.-E., Koo J., Kim T.H., Park J.-E., Choi K.-C. (2023). TGF-β2 antisense oligonucleotide enhances T-cell mediated anti-tumor activities by IL-2 via attenuation of fibrotic reaction in a humanized mouse model of pancreatic ductal adenocarcinoma. Biomed. Pharmacother..

[47] Sun L., Wang J., Wang Q., He Z., Sun T., Yao Y., Wang W., Shen P. (2022). Pretreatment of umbilical cord derived MSCs with IFN-γ and TNF-α enhances the tumor-suppressive effect on acute myeloid leukemia. Biochem. Pharmacol..

[48] Berglund L.J., Avery D.T., Ma C.S., Moens L., Deenick E.K., Bustamante J., Boisson-Dupuis S., Wong M., Adelstein S., Arkwright P.D. (2013). IL-21 signalling via STAT3 primes human naïve B cells to respond to IL-2 to enhance their differentiation into plasmablasts. Blood.

[49] Gui L., Zeng Q., Xu Z., Zhang H., Qin S., Liu C., Xu C., Qian Z., Zhang S., Huang S. (2016). IL-2, IL-4, IFN-γ or TNF-α enhances BAFF-stimulated cell viability and survival by activating Erk1/2 and S6K1 pathways in neoplastic B-lymphoid cells. Cytokine.

[50] Wang R., Hua Y., Wu H., Wang J., Xiao Y.-c., Chen X., Ao Q., Zeng Q., Zhu X., Zhang X. (2023). Hydroxyapatite nanoparticles promote TLR4 agonist-mediated anti-tumor immunity through synergically enhanced macrophage polarization. Acta Biomater..

[51] Li G.-Y., Feng Y.-Q., Jia Y.-F., Wang K.-F., Li Y., Zhang S.-J., Han S.-X., Wang J.-C. (2023). Metformin enhances T lymphocyte anti-tumor immunity by increasing the infiltration via vessel normalization. Eur. J. Pharmacol..

[52] Song A., Ding T., Wei N., Yang J., Ma M., Zheng S., Jin H. (2023). Schisandrin B induces HepG2 cells pyroptosis by activating NK cells mediated anti-tumor immunity. Toxicol. Appl. Pharmacol..

[53] Fu J., Li T., Yang Y., Jiang L., Wang W., Fu L., Zhu Y., Hao Y. (2021). Activatable nanomedicine for overcoming hypoxia-induced resistance to chemotherapy and inhibiting tumor growth by inducing collaborative apoptosis and ferroptosis in solid tumors. Biomaterials.

[54] Liu S., Jiang X., Shang Z., Ji Y., Wang H., Wang Z., Wang P., Zhang Y., Xiao H. (2020). N-glycan structures of target cancer biomarker characterized by two-dimensional gel electrophoresis and mass spectrometry. Anal. Chim. Acta.

[55] Yang B., Xiao B., Sun T. (2013). Antitumor and immunomodulatory activity of Astragalus membranaceus polysaccharides in H22 tumor-bearing mice. Int. J. Biol. Macromol..

[56] Zheng Y., Wang W.-d., Li Y. (2015). Antitumor and immunomodulatory activity of polysaccharide isolated from Trametes orientalis. Carbohydr. Polym..

[57] Ji H., Fan Y., Gao X., Gong Y., Dai K., Wang Z., Xu B., Yu J. (2023). The Protective Effects of Water-Soluble Alginic Acid on the N-Terminal of Thymopentin. Molecules.

[58] Iraporda C., Errea A., Romanin D.E., Cayet D., Pereyra E., Pignataro O., Sirard J.C., Garrote G.L., Abraham A.G., Rumbo M. (2015). Lactate and short chain fatty acids produced by microbial fermentation downregulate proinflammatory responses in intestinal epithelial cells and myeloid cells. Immunobiology.

[59] Ji H.-y., Liu C., Dai K.-y., Yu J., Liu A.-j., Chen Y.-f. (2021). The immunosuppressive effects of low molecular weight chitosan on thymopentin-activated mice bearing H22 solid tumors. Int. Immunopharmacol..

[60] Yu J., Ji H.-y., Liu C., Liu A.-j. (2020). The structural characteristics of an acid-soluble polysaccharide from Grifola frondosa and its antitumor effects on H22-bearing mice. Int. J. Biol. Macromol..

